# Analysis of Human Gut Microbiome: Taxonomy and Metabolic Functions in Thai Adults

**DOI:** 10.3390/genes12030331

**Published:** 2021-02-25

**Authors:** Nachon Raethong, Massalin Nakphaichit, Narissara Suratannon, Witida Sathitkowitchai, Wanlapa Weerapakorn, Suttipun Keawsompong, Wanwipa Vongsangnak

**Affiliations:** 1 Department of Zoology, Faculty of Science, Kasetsart University, Bangkok 10900, Thailand; nachonase@hotmail.com; 2 Department of Biotechnology, Faculty of Agro-Industry, Kasetsart University, Bangkok 10900, Thailand; fagimln@ku.ac.th (M.N.); witidas@yahoo.com (W.S.); suttipun.k@ku.ac.th (S.K.); 3 Center for Advanced Studies for Agriculture and Food, Kasetsart University Institute for Advanced Studies, Kasetsart University, (CASAF, NRU-KU), Bangkok 10900, Thailand; 4 Pediatric Allergy & Clinical Immunology Research Unit, Division of Allergy and Immunology, Department of Pediatrics, Faculty of Medicine, Chulalongkorn University, King Chulalongkorn Memorial Hospital, The Thai Red Cross Society, Bangkok 10330, Thailand; narissara.su@chula.ac.th (N.S.); wparw@yahoo.com (W.W.); 5 Microarray Research Team, National Center for Genetic Engineering and Biotechnology, 113 Thailand Science Park, Phahonyothin Road, Khlong Luang, Pathum Thani 12120, Thailand; 6 Omics Center for Agriculture, Bioresources, Food, and Health, Kasetsart University (OmiKU), Bangkok 10900, Thailand

**Keywords:** gut microbiome, Thai adults, whole metagenome shotgun (WMGS) sequencing, meta-gene catalogue, metabolic functions

## Abstract

The gut microbiome plays a major role in the maintenance of human health. Characterizing the taxonomy and metabolic functions of the human gut microbiome is necessary for enhancing health. Here, we analyzed the metagenomic sequencing, assembly and construction of a meta-gene catalogue of the human gut microbiome with the overall aim of investigating the taxonomy and metabolic functions of the gut microbiome in Thai adults. As a result, the integrative analysis of 16S rRNA gene and whole metagenome shotgun (WMGS) sequencing data revealed that the dominant gut bacterial families were *Lachnospiraceae* and *Ruminococcaceae* of the Firmicutes phylum. Consistently, across 3.8 million (M) genes annotated from 163.5 gigabases (Gb) of WMGS sequencing data, a significant number of genes associated with carbohydrate metabolism of the dominant bacterial families were identified. Further identification of bacterial community-wide metabolic functions promisingly highlighted the importance of *Roseburia* and *Faecalibacterium* involvement in central carbon metabolism, sugar utilization and metabolism towards butyrate biosynthesis. This work presents an initial study of shotgun metagenomics in a Thai population-based cohort in a developing Southeast Asian country.

## 1. Introduction

The human gut ecosystem is extremely large and harbors hundreds of microbiome [1] with over three million genes encoded by their collective genomes, which accounts for 150 times more than the human gene complement [2]. Therefore, it is not surprising that many specific genes, which are lacking in the human genome, perform a role in the gut microbiome where they facilitate host metabolism and have the capacity to retain energy and nutrients from the diet through extraordinary metabolic interactions that exchange metabolites across a number of microbial species (∼570 species) and host cells in the gut environment [3,4]. Moreover, the microbiome plays an essential role in human immune maturation by mediating host immune responses [5]. Both host and environmental factors can influence microbial colonization and functions. Several bacterial taxa, such as *Prevotella* and *Bifidobacterium,* which predominantly colonize in healthy humans, together with other microbial members of the gut, such as *Blautia* and *Roseburia* species, synthesize short-chain fatty acids (SCFA) including acetate, propionate and butyrate [6]. These microbial-derived SCFA are subsequently taken up by the host as an energy source [7]. In particular, butyrate has been found to suppress inflammatory and allergic responses to food antigens by mediating the differentiation of colonic regulatory T cells in the colon [8]. Besides, it has been observed that some microbial members are capable of producing vitamins and neurotransmitters, such as γ-aminobutyric acid (GABA), with sufficient amounts in the human gut [9,10]. Thus, the diversity of gut microbiome and their functions are considerably associated with host nutrition and health status whereas dysfunctions of the gut microbiome can predispose the host to a number of human diseases, such as diabetes [11], inflammatory bowel disease [12], cardiovascular disease [13] and respiratory illness [14]. 

Therefore, understanding the fitness and features of the human gut microbiome has become an important area of research, which could provide an alternative therapeutic avenue for the relevant co-morbidities [14]. In recent years, next generation sequencing based on 16S rRNA gene sequencing and shotgun sequencing has become more feasible, not only to obtain taxonomic information, but also to assess the functional attributes of the human gut microbiome [15,16]. A recent metagenomic study of a cohort of immigrants in the United States (US) revealed that the enrichment of the fiber-degrading ability of the gut microbiome in healthy Hmong was remarkably associated with diet. This highlighted the prevalence of plant-based ingredients in their traditional foods during pre-immigration in Northern Thailand. It has been noted that the diversity and richness of fiber-degrading bacteria in Hmong gut microbiome are the highest in Thailand, and decrease according to prolonged American diet acculturation after relocation to the US [17]. Loss of native gut microbiome has remarkably increased the risk of obesity and other chronic diseases in US immigrants compared to the Hmong people who currently live in Thailand [17]. Regarding Thai populations, the effects of different dietary habits and health conditions on the composition of gut microbiota have been previously investigated in different Thai cohorts of children, adults and elderly persons [18,19,20,21,22]. However, these analyses were only based on 16S rRNA gene sequencing data [23] and could not provide the full taxonomic information and metabolic function of gut microbiome. 

Therefore, this study aimed to investigate the taxonomic profiles and to annotate metabolic functions of the gut microbiome of Thai adults using integrative 16S rRNA gene and whole metagenome shotgun (WMGS) sequencing data. The 16S rRNA gene and WMGS sequencing data were initially obtained from the gut microbiome of Thai adults by DNA extraction from fecal samples, and then run through Illumina sequencing. After that, the sequencing data were processed through different bioinformatics tools and databases for analysis of the taxonomic profiles of gut microbiome. Then, metagenome annotation was performed for the construction of a meta-gene catalogue of Thai gut microbiome. These were used to further identify the metabolic functions of gut microbiome of Thai adults. This study serves as a framework for bacterial community-wide metabolic functional studies of the gut microbiome. Our work presents an initial study of shotgun metagenomics in a Thai population-based cohort in a developing Southeast Asian country.

## 2. Results and Discussion

Among 60 Thai adults from the middle region of Thailand that were enrolled in the cohort, 56 participants were selected based on stringent inclusion and exclusion criteria (see Methods) for further assessment and analysis. Briefly, they were well-characterized in terms of their physical and cognitive health status with regular diets. As summarized in Supplementary Materials, Table S1, the gender ratio of the studied cohort was 0.3:1 (male: female) and the mean age and body mass index (BMI) of all participants was 30.0 ± 5.2 years and 21.3 ± 2.0 kg/m^2^, respectively. In addition, the daily energy and nutrient intakes estimated from the recorded food intake of all participants, as listed in Tables S2 and S3, were consistent with the dietary recommendations for young Thai adults from Ivanovitch et al. (2014) [24]. 

### 2.1. Assessment of Taxonomic Profiles of Gut Microbiome from Thai Adults Using 16S rRNA Gene Sequencing Data

**Figure 1:**
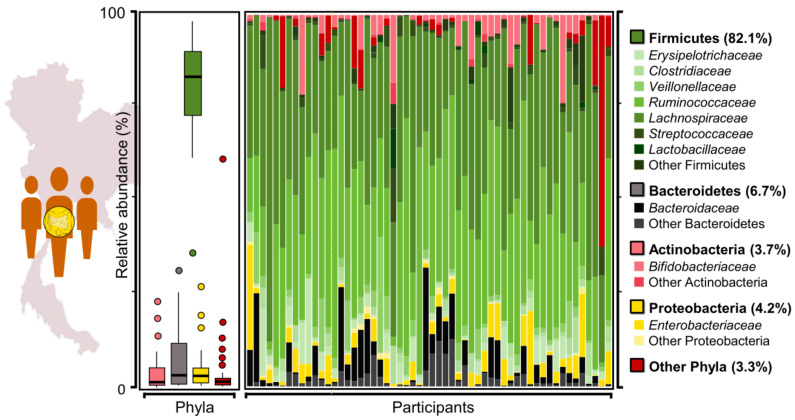
The taxonomic profiles of gut microbiome from Thai adults using 16S rRNA gene sequencing data.

To assess the taxonomic profiles of gut microbiome from 56 participants, 16S rRNA gene sequencing was initially performed. As presented in Figure 1, interestingly, we found that four phyla, i.e., Firmicutes, Bacteroidetes, Actinobacteria and Proteobacteria were most commonly identified. Among these four phyla, Firmicutes showed the highest relative abundance of bacterial community, accounting for 82.1%, of which the order Clostridiales, belonging to the class Clostridia in the phylum Firmicutes was dominant with high abundances (>25%). Considering the majority of the samples from the 56 participants and the top ten bacterial families (see Table S4), we found two dominant families, namely, *Lachnospiraceae* and *Ruminococcaceae* in Thai adults. These two families are consistent with the findings of La-ongkham et al. (2020) [22], whose study focused on the core taxonomic feature of the Thai gut microbiome. 

Furthermore, a comparative analysis of taxonomic profiles of the gut microbiome among Thai adults from this study, Hmong adults and American adults in the US [17] was performed using 16S rRNA gene sequencing data and literature surveys. As a result, we found that the high relative abundance of *Ruminococcaceae* in Thai adults was similar to Hmong adults who were currently living in Thailand whereas the lowest abundance of *Ruminococcaceae* was observed in Hmong and American adults who were currently living in the US [17]. In addition, it was noticed that the high relative abundances of *Lachnospiraceae* and *Ruminococcaceae* in Thai adults were consistent with many previous reports in other Asian cohorts, i.e., Japanese [25], Chinese [26] and Indonesian [27]. 

Moreover, the association analysis between demographic and clinical characteristics in the cohort (e.g., age, gender and BMI), as well as the relative abundance of the dominant bacterial families was performed using Spearman’s rank correlation (Table S5). As a result, we found a weak association between the abundance of *Ruminococcaceae* and BMI (Figure S1). As observed, the higher abundance of *Ruminococcaceae* was found with lower BMI. This finding is in agreement with other studies that have shown an association between *Ruminococcaceae* and a lower risk of weight gain in a Caucasian cohort of volunteer adult twins from the United Kingdom through enhanced carbohydrate and energy metabolism [28,29]. Accordingly, this suggests that the identification of bacterial community-wide metabolic functions should be further performed. 

The remaining phyla showed low relative abundances in the bacterial community, accounting for 6.7, 3.7, 4.2, and 3.3% of Bacteroidetes, Actinobacteria, Proteobacteria and others, respectively. 

### 2.2. Construction of Meta-Gene Catalogue of Thai Gut Microbiome from WMGS Sequencing Data

**Figure 2:**
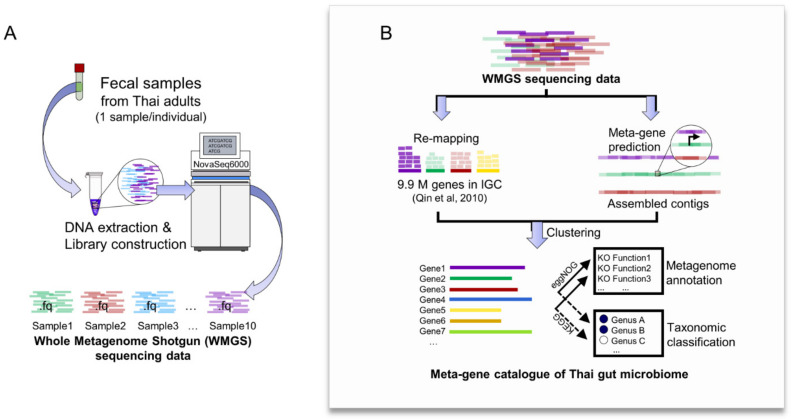
Schematic overview of whole metagenome shotgun (WMGS) sequencing assessment and meta-gene catalogue of Thai gut microbiome construction. (**A**) Assessment of WMGS sequencing data obtained from the gut microbiome of Thai adults and (**B**) An integrated pipeline for constructing the meta-gene catalogue of Thai gut microbiome.

As the gut microbiome exhibits a number of bacterial species that are genetically diverse, and therefore, contain different sets of metabolic functions and pathways, it was of interest to investigate the functional roles of the gut microbiome of Thai adults. However, because of the limitations of the 16S rRNA gene sequencing method, which does not sequence a specific functional gene directly, the metabolic functional annotation of genes and pathways of the microbiome can only be implied based on the taxonomic inferences [30]. Thus, the WMGS sequencing method, which is a powerful culture-independent method for annotating meta-genes and functions of the complex microbial communities, was selected in this study. The schematic overview of the WMGS sequencing assessment is illustrated in Figure 2A. Initially, the metagenomic DNA isolated from the fecal samples of ten participants selected from the 56 participants were sequenced and raw reads were obtained at an average depth of 55.0 ± 8.8 million (M) paired-end reads per sample. After removing adaptors and low-quality sequences, as well as human genomic contaminants, the clean data were finally retrieved with the percentage quality of sequences accounting for 99.1 ± 0.4% on average (Table S6). 

Once all the clean reads obtained from each sample were combined, a large fraction of 163.5 Gb was acquired and processed through an integrated pipeline for constructing the meta-gene catalogue of Thai gut microbiome (Figure 2B). This resulted in the identification of a total of 3.8 million (M) genes from the metagenomics of Thai gut microbiome, which comprises 1.7 M newly predicted genes (see Methods, Section 4.5) and 2.1 M genes homologous with the global reference gene catalogue of human gut microbiome [2]. Taken together, the meta-gene catalogue of Thai gut microbiome is available at https://github.com/sysbiomics/meta-gene (accessed on 19 February 2021).

### 2.3. Annotation of Genes Associated with Metabolic Functions of Thai Gut Microbiome from WMGS Sequencing Data

**Figure 3:**
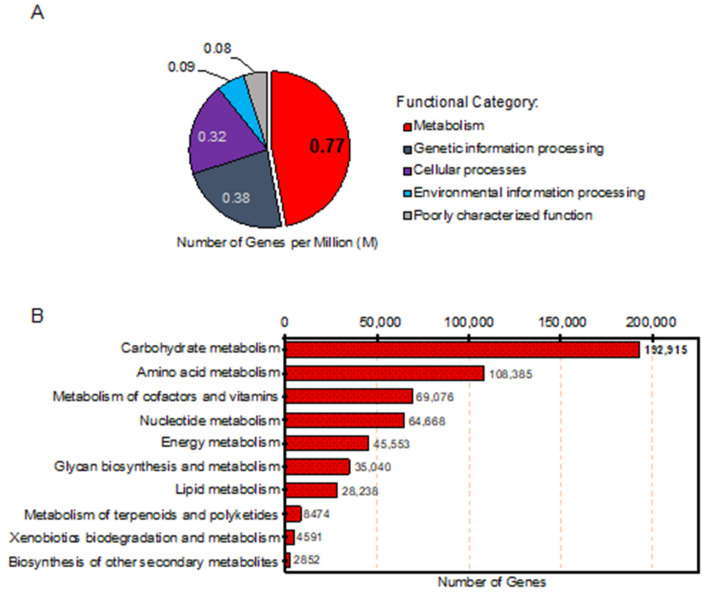
Number of genes involved in metabolic functions of Thai gut microbiome. (**A**) The distribution of genes across the five main functional categories, and (**B**) The number of genes involved in metabolic functions across the different metabolic pathways.

To annotate genes associated with the metabolic functions of the gut microbiome in Thai adults, a total of 3.8 M genes in the meta-gene catalogue of Thai gut microbiome were searched against the KEGG database. As a result, there were 1.6 M genes classified to the KEGG orthology (KO), which distributed them into five functional categories including metabolism (0.77 M genes), genetic information processing (0.38 M genes), cellular processes (0.32 M genes), environmental information processing (0.09 M genes) and poorly characterized functions (0.08 M genes), as shown in Figure 3A. 

Considering the major category, 559,792 genes out of 0.77 M genes that belonged to the functional category of metabolism, were annotated with metabolic functions involved in metabolism of carbohydrates (192,915 genes), amino acids (108,385 genes), cofactors and vitamins (69,076 genes), nucleotide (64,668 genes), energy (45,553 genes), glycan (35,040 genes), lipid (28,238 genes) and other metabolites, e.g., terpenoids, polyketides and secondary metabolites (15,917 genes), as illustrated in Figure 3B. The results clearly showed that the highest number of genes were involved in carbohydrate metabolism of the gut microbiome in Thai adults. In addition, a comparative analysis of the meta-gene catalogues of the gut microbiome at the functional level, between Thai adults from this study and multiple populations from China, European countries and US was performed [2]. According to KEGG, the results showed that a total of 66 KOs were uniquely found in Thai adults, as listed in Table S7. Among these KOs, the number of enzymes involved in carbohydrate metabolism were identified, for example, sorbose reductase (EC: 1.1.1.289) and 1,5-anhydro-D-fructose reductase (EC: 1.1.1.263). As observed, the results are in agreement with earlier shotgun metaproteomic studies in Sardinian and Swedish cohorts [31,32,33]. 

### 2.4. Bacterial Community-Wide Metabolic Functional Analysis Involved in Carbohydrate Metabolism 

**Figure 4:**
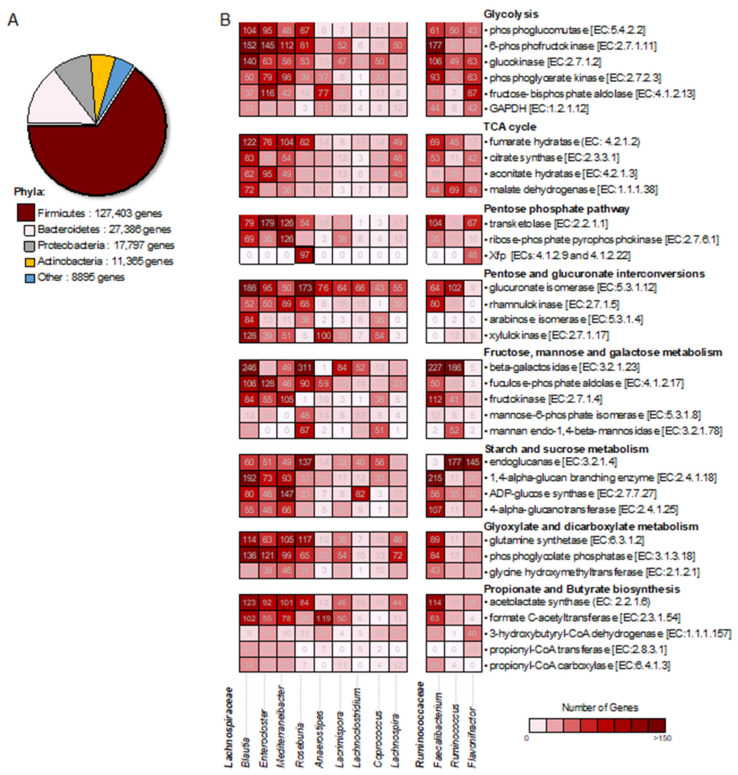
Bacterial community-wide metabolic functional analysis involved in carbohydrate metabolism. (**A**) Total number of genes involved in carbohydrate metabolism across the different phyla, and (**B**) Distribution of genes involved in metabolic functions and pathways involved in carbohydrate metabolism across taxonomic profile of bacterial community. Abbreviated enzyme names are as follows: GAPDH, glyceraldehyde 3-phosphate dehydrogenase; Xfp xylulose-5-phosphate/fructose-6-phosphate phosphoketolase.

To further assess the taxonomic profiles of the gut microbiome involved in carbohydrate metabolism, the analysis of bacterial community-wide metabolic functions was thoroughly applied to 192,915 genes involved in carbohydrate metabolism. As a result, 99.93% of genes were taxonomically assigned to Firmicutes, Bacteroidetes, Proteobacteria and Actinobacteria, as depicted in Figure 4A. Among these phyla, Firmicutes displayed a significant number of genes (127,403 genes), which accounted for 66.0% of carbohydrate metabolism, of which 98,874 out of 127,403 genes were identified in the class of Clostridia. This result highlighted how to best find the families, genus or species level and their functional roles in carbohydrate metabolism. As a result of the utility of the bacterial community-wide metabolic functional analysis, a total of 98,874 genes involved in carbohydrate metabolism were grouped according to their taxonomic lineages at genus level and metabolic functions/pathways. Accordingly, as schematized in Figure 4B, two families across 12 genera were identified. Promisingly, nine genera in *Lachnospiraceae* and three genera in *Ruminococcaceae* were identified with a high number of genes involved in carbohydrate metabolism under the threshold of 10,000 genes/family and 2000 genes/genus. Furthermore, metabolic functions were associated with central carbon metabolism (e.g., glycolysis, TCA (tricarboxylic acid) cycle and pentose-phosphate pathway), sugar metabolism and utilization (e.g., pentoses, fructose, mannose, galactose, starch, and sucrose), glyoxylate and dicarboxylate metabolism, and propionate and butyrate biosynthesis.

More interestingly, the high number of gene functions identified among butyrate-producing bacteria, such as *Blautia, Enterocloster, Mediterraneibacter* (*Ruminococcus torques*) and *Faecalibacterium*, include glucokinase (EC: 2.7.1.2), 6-phosphofructokinase (EC: 2.7.1.11), phosphoglucomutase (EC: 5.4.2.2), phosphoglycerate kinase (EC: 2.7.2.3), formate C-acetyltransferase (EC: 2.3.1.54), acetolactate synthase (EC: 2.2.1.6), and many other enzymes responsible for the essential steps of glycolysis and propionate and butyrate biosynthesis (Table S8). These observed results were consistent with the metabolic traits of butyrate-producing bacteria for butyrate production, which are normally identified in gut microbiome in healthy adults [32]. 

Moreover, we also found that *Roseburia* exhibited a high number of genes involved in xylulose-5-phosphate/fructose-6-phosphate phosphoketolase (Xfp, ECs: 4.1.2.9 and 4.1.2.22), which is the key catalytic enzyme in the hemicellulosic complex oligosaccharides fermentation pathway [34]. This finding is consistent with the most recent genome-scale metabolic reconstruction of the carbohydrate degradation and utilization pathways of *Roseburia*, which demonstrated the ability of *Roseburia* species to ferment pentoses (e.g., xylose and arabinose) and oligosaccharides (e.g., xylooligosaccharides, arabinoxylans and arabinogalactans) as carbon sources for growth through Xfp [35]. Additionally, it has been noticed that Xfp plays a pivotal role in intermediary carbohydrate metabolism, providing an efficient way of generating acetyl-CoA and acetate when grown on pentose as the sole carbon source [36]. Here, acetyl-CoA and acetate are key metabolites for dietary carbohydrate and SCFA metabolism, which have been shown to be important for the growth and butyrate production of the gut microbiome, e.g., *Faecalibacterium* [37]. This result suggests that bacterial community-wide metabolic functions are key for gut health through providing preferential metabolic precursors, e.g., acetyl-CoA and acetate for butyrate production.

## 3. Conclusions

Our Illumina-based metagenomic data were obtained by using 16S rRNA gene and WMGS sequencing methods and were analyzed through the integration of bioinformatics and a systems biology approach, which successfully identified the number of genes involved in carbohydrate metabolism of the gut microbiome in Thai adults, and showed they belonged to dominant families, e.g., *Lachnospiraceae* and *Ruminococcaceae* of the Firmicutes phylum. Further identification of bacterial community-wide metabolic functions highlighted the importance of *Roseburia* and *Faecalibacterium* involvement in central carbon metabolism, sugar utilization and metabolism for butyrate biosynthesis. Future potential applications of microbiome-based large-scale datasets to determine microbial community-wide metabolic functions are therefore needed to shed further light on the gut-microbiome-metabolic axis implicated in human health. 

## 4. Materials and Methods

### 4.1. Participants and Fecal Sample Collection

Sixty Thai adults living in Bangkok and near the capital city of Thailand, aged between 18–45 years and with a BMI of 18.5–24.0 kg/m^2^ were enrolled in the cohort. It is worth noting that this study was initially expected to have a high number of participants under stringent inclusion and exclusion criteria for age, health status and dietary intake. Of 60 Thai adults, 56 participants hereby completed the study under criteria as summarized in Table S1. Briefly, all participants (non-smoker with regular bowel habits including normal defecation frequency) were recruited at King Chulalongkorn Memorial Hospital, Bangkok, Thailand in 2019. All participants had no history of intestinal diseases and diarrhea in the months prior to sampling as well as no family history of colorectal cancer. In addition, none of them had received antibiotics within at least three months as well as probiotics, prebiotics and synbiotics within one month before fecal sample collection. Participants with food intolerance or allergy to coconut were also excluded. This study was approved by the Thai Clinical Trials Registry under the trial identification number TCTR20190426003 and the Ethics Committee of King Chulalongkorn Memorial Hospital, Bangkok, Thailand (IRB No. 388/61). All methods were performed in accordance with the relevant guidelines and regulations. Written consent was obtained from all participants. Apart from the demographic and clinical characteristics, dietary information was also collected from all participants by a dietary record questionnaire form that inquired about their menu and food ingredients. The INMUCAL-Nutrients V.4.0, Database version NB.4 was used for estimating the energy (kcal/day) and nutrient content (g, mg, or µg) of the recorded foods of the participants before the fecal sample collection. As detailed in Table S2, the average total energy expenditure from the recorded foods of all participants (1400.90 ± 736.17 kcal/day) was consistent with the energy intake of a sample of Thai sedentary workers in the Bangkok city area, aged 20–50 years (1428  and 1485 kcal/day for females and males, respectively) [24]. In particular, the energy intake from carbohydrate (48.64 ± 10.77%), protein (19.05 ± 6.79%) and total fat (32.31 ± 8.58%) were similar across the studied cohort at the time of sampling (Table S3). Notably, all participants did not consume excessive alcohol (<3 drinks per day). For sample collection, fresh fecal was collected and placed into the collection tube and kept it in a cooler bag, immediately at the time of defecation. Then, they were delivered to the laboratory and stored at −80 °C.

### 4.2. Metagenomic DNA Extraction

The 56 fecal samples were prepared for metagenomic DNA extraction according to the modified method of Nakphaichit et al. (2014) [38]. Briefly, the fecal samples were centrifuged at 13,000× *g* for 2 min and supernatant was discarded. The remaining pellet was washed twice by 1 mL phosphate-buffered saline solution (PBS) with centrifugation at 13,000× *g* for 5 min and subsequently suspended with 900 µL PBS. The total DNA was extracted and purified from the suspension sample by the magnetic bead-based method using a bead meter and QIAamp DNA Stool Mini Kit (Qiagen, Hilden, Germany), respectively, and stored at −20 °C.

### 4.3. 16S rRNA Gene Sequencing, Reads Processing and Microbial Composition Analysis

The variable regions of V3-V4 of 16S rRNA gene from a total of 56 metagenomic DNA samples were amplified by forward and reverse primers, i.e., Imina-V3-V4-F (5′-TCGTCGG CAGCGTCAGATGTGTATAAGAGACAGCCTACGGGNGGCWGCAG-3′) and Imina-V3-V4-R (5′-GTCTCGTGGGCTCGGAGATGTGTATAAGAGACAGGACTACTACHVG GGTATCTAATCC-3′) with cycling conditions of initial denaturation of 94 °C for 2 min, followed by 25 cycles of denaturation at 94 °C for 20 s, annealing at 57 °C for 30 s, extension at 72 °C for 30 s and a final extension at 72 °C for 10 min. The amplified products were then purified using NucleoSpin Gel and PCR Clean-up (MACHEREY-NAGEL Inc., USA) according to the manufacturer’s protocol and sequenced by the Illumina MiSeq platform at Omics Sciences and Bioinformatics Center (Chulalongkorn University, Thailand). Raw sequencing pair-end reads were subjected to quality filtering using BBDuk program in the BBTools (Bushnell B.—sourceforge.net/projects/bbmap) and the primer at the 5′ was removed using seqtk (https://github.com/lh3/seqtk (accessed on: 25 November 2020)). After chimera removal through the DADA2 pipeline [39], the high-quality clean reads were obtained with an average of 41,843 ± 10,553 reads per sample and subjected for taxonomy identification by QIIME 2 (version 2019.1) [40] using Greengenes version 13.8 as a bacterial/archaeal 16S rRNA gene sequence database [41]. The cut-off confidence was indicated at 0.7. 

For association analysis between demographic and clinical characteristics of the study cohort (i.e., age, gender and BMI), as well as the relative abundance of the dominant bacterial taxa across the 56 participants, the Spearman’s rank correlation was applied using the “cor.test” command in the “stats” package version 4.0.2 in R. Using a threshold (*p* < 0.05), the strong, the moderate and the weak associations between the variables were defined by the absolute value of the correlation coefficient (*r*) ranges of 0.5–1.0, 0.3–0.49 and 0.1–0.29, respectively [22]. A scatter plot was used to display the correlation between the two variables (i.e., relative abundance and BMI), which was generated by using the “ggscatter” command in the “ggpubr” package version 0.4.0 in R.

### 4.4. Assessment of Whole Metagenome Shotgun (WMGS) Sequencing Data Obtained from the Gut Microbiome of Thai Adults

The metagenomic DNA isolated from the fecal samples of ten participants selected from the 56 participants were purified and they underwent quality control assessments including: (1) DNA purity test by Nanodrop (OD260/OD280), (2) DNA degradation and potential contamination test by agarose gel electrophoresis, and (3) DNA quantification by Qubit 2.0. Afterwards, the qualified DNA samples were sheared into fragments by restriction enzyme and then ligated with adapter for NEBNext library preparation for Illumina sequencing. The genomic DNA libraries were generated and subsequently sequenced with paired-end mode of 150 base pairs (2 × 150 bps) by Illumina NovaSeq6000 System at Novogene (Hong Kong, China) as shown in Figure 2A. Raw sequencing data were subjected to quality filtering and a human genome removal process using BBDuk and BBMap programs in the BBTools (Bushnell B.—sourceforge.net/projects/bbmap). The clean reads unmapped to human genome were yielded as the WMGS sequencing data from each sample. 

### 4.5. An Integrated Pipeline for Constructing the Meta-Gene Catalogue of Thai Gut Microbiome from WMGS Sequencing Data

In order to obtain virtually all of the prevalent gut microbial genes in Thai adults, the WMGS sequencing data were processed through an integrated pipeline for constructing the meta-gene catalogue of Thai gut microbiome (Figure 2B). This integrated pipeline was developed based on the reference-based and de novo assembly-based methods. For the reference-based method, the WMGS sequencing data were mapped against the integrated gene catalogue (IGC) of human gut microbiome, which comprised of 9,879,896 genes in total, according to previously described method using BWA-MEM and SAMtools [2,42,43,44], in which only genes mapped with WMGS sequencing data were retrieved. For the latter method, the WMGS sequencing data were subjected to the de novo assembly process by MEGAHIT version 1.2.9 [45] and meta-gene prediction. Briefly, the assembly of metagenomic datasets, i.e., assembled contigs larger than 500 bps of gut microbiome of individual samples was performed for gene prediction by Prodigal version 2.6.3 [46], in which, the predicted gene length higher than 100 bps from all individuals were retrieved. Then, overall retrieved genes from the two methods were combined and redundant genes were eliminated by clustering using CD-HIT version 4.8.1 with a 95% sequence identity [47]. Finally, the meta-gene catalogue of the Thai gut microbiome was obtained and subjected to functional annotation and taxonomic assignment by eggNOG-mapper version 2.0.0 [48,49] and GhostKOALA [50], respectively (Figure 2B).

## Data Availability

Raw sequencing data are available in the National Center for Biotechnology Information Sequence Read Archive (NCBI-SRA) repository under the BioProject accession number PRJNA637175.

## Supplementary Materials

The following are available online at https://www.mdpi.com/2073-4425/12/3/331/s1, Supplementary Figure S1: Spearman’s correlation between the abundance of *Ruminococcaceae* and BMI of 56 Thai adults. Supplementary Table S1: Demographic characteristics of 56 Thai adults participating in the study cohort. Supplementary Table S2: The energy and nutrient contents of the recorded foods of 56 Thai adults. Supplementary Table S3: The energy distribution of the recorded foods of 56 Thai adults. Supplementary Table S4: The relative abundances of the 10 dominant bacterial families identified in the fecal samples of 56 Thai adults. Supplementary Table S5: Spearman’s correlation between the abundance of the dominant bacterial families and demographic and clinical characteristics of 56 Thai adults. Supplementary Table S6: Whole metagenome shotgun (WMGS) sequencing data of the ten selected samples out of 56 Thai adults. Supplementary Table S7: The list of unique KOs for the meta-gene catalogue of Thai gut microbiome. Supplementary Table S8: Bacterial community-wide metabolic functional analysis of gut microbiome involved in carbohydrate metabolism.
