# Supplementary material for: Analysis of Human Gut Microbiome: Taxonomy and Metabolic Functions in Thai Adults
Source: Genes (Basel). 2021 Feb 25;12(3):331. doi: 10.3390/genes12030331 (PMC7996147; doi:10.3390/genes12030331)

Supplementary Figure S1: Spearman's correlation between the abundance of *Ruminococcaceae* and BMI of 56 Thai adults.

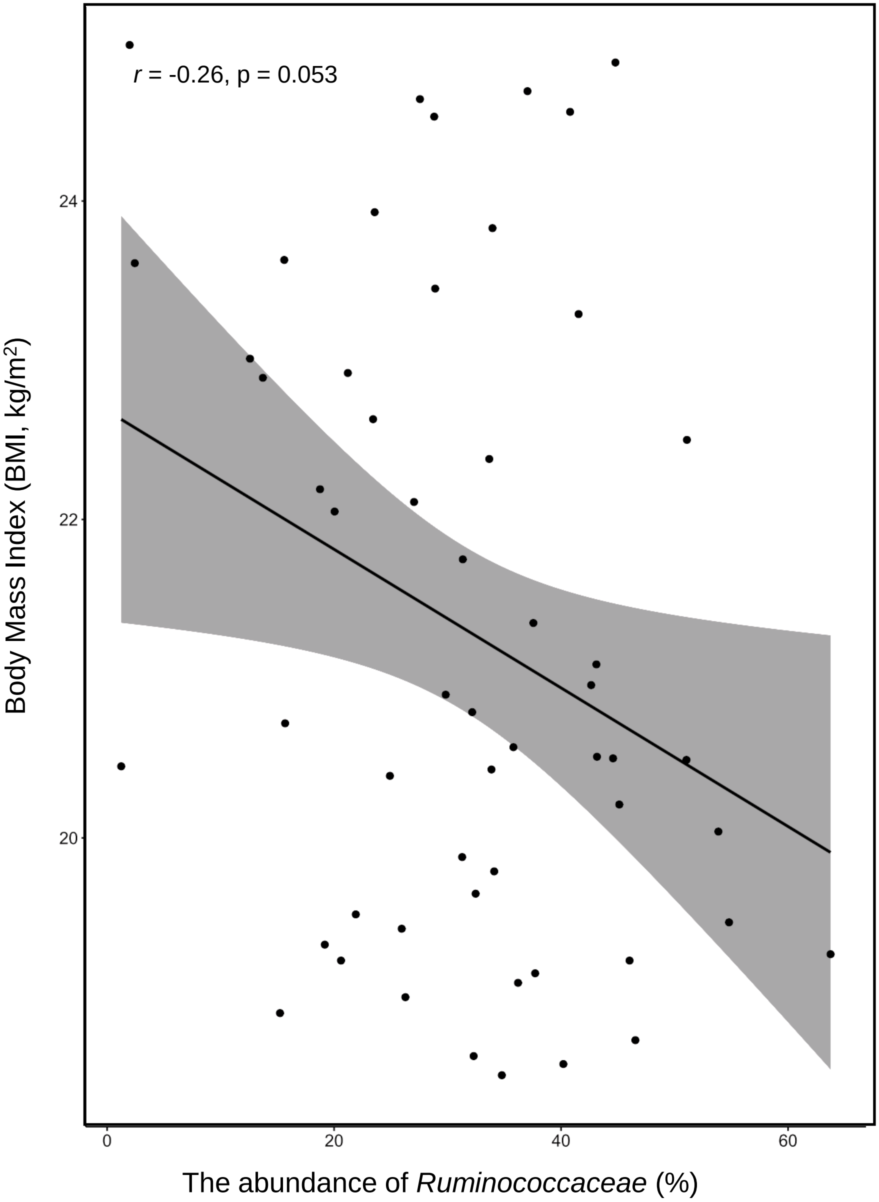

Supplement: Supplementary file 1 [file genes-12-00331-s001.zip › Supplementary_files/FigureS1.pdf]
